# Goldenhar Syndrome—Embryological, Anatomical, Etiopathogenic Mechanisms and Treatment of Obstructive Sleep Apnea

**DOI:** 10.3390/ijms27146169

**Published:** 2026-07-10

**Authors:** Katarzyna Sluzalec-Wieckiewicz, Edward Kijak, Irena Dus-Ilnicka, Marcin Mikulewicz, Joanna Laskowska, Piotr Seweryn, Conrad Maslowiec, Lucia Miralles-Jorda, Marta Mazur, Anna Paradowska-Stolarz

**Affiliations:** 1 Department of Prosthodontics, Wrocław Medical University, ul. Krakowska 26, 50425 Wroclaw, Poland; katarzyna.sluzalec-wieckiewicz@umw.edu.pl (K.S.-W.);; 2 Division of General and Experimental Pathology, Department of Clinical and Experimental Pathology, Wroclaw Medical University, ul. T. Marcinkowskiego 1, 50-368 Wroclaw, Poland; 3 Division of Facial Abnormalities, Department of Maxillofacial Orthopedics and Orthodontics, Faculty of Dentistry, Wroclaw Medical University, Krakowska 26, 50-425 Wroclaw, Poland; 4 Department of Experimental Dentistry, Wroclaw Medical University, Krakowska 26, 50-425 Wroclaw, Poland; 5 University Dental Center, Krakowska 26, 50-425 Wroclaw, Poland; 6 Department of Dentistry, Catholic University of Valencia, Quevedo Str. 2, 46001 Valencia, Spain; 7 Interdisciplinary Department of Wellbeing, Health and Environmental Sustainability-BeSSA Department, Sapienza University of Rome, 02100 Rieti, Italy

**Keywords:** OSA, obstructive sleep apnea, Goldenhar syndrome, ocular–auricular–vertebral syndrome, 22q chromosome, congenital disorder, breathing disorder

## Abstract

Goldenhar syndrome is a developmental defect of the 1st and 2nd brachial arches. Facial asymmetry and ear–eye abnormalities are observed. Frequent presence of concomitant issues, including severe OSA (obstructive sleep apnea) and sialorrhea, have also been recorded. The presentation of Goldenhar syndrome is highly variable, and treatment procedures also differ, with surgical treatment often being mandatory. Common symptoms of OSA include loud snoring, gasping, and morning headaches. If untreated, it may lead to high blood pressure, hyperinsulinemia, heart problems, stroke, and daytime sleepiness. Its etiology in Goldenhar syndrome is primarily anatomical and multifactorial. The most common treatment options are based on surgical procedures. Continuous positive airway pressure (CPAP) could be considered for use in individuals with Goldenhar syndrome. Mandibular advancement is a standard procedure for relieving the symptoms of OSA. In general, mandibular advancement would reduce apnea and hypopnea. Another method of managing OSA is hypoglossal nerve stimulation (HNS). Adenotonsillectomy is often the first-line surgical treatment for OSA but it is rarely curative in Goldenhar patients due to underlying skeletal issues. In contrast to American Academy of Sleep Science recommendations of managing OSA, in patients with congenital syndromes, surgical intervention seems to be the most optimal method of treatment.

## 1. Introduction

Goldenhar syndrome is a developmental defect of the 1st and 2nd brachial arches. It is also known as OAVS (ocular–auricular–vertebral syndrome). It arises from a combination of genetic and environmental factors (with the 22q chromosome predominating in the etiology) [1,2,3]. Goldenhar syndrome is one of the most common congenital syndromes exhibiting facial asymmetry and affects roughly 3500 to 5000 newborns [4]. The syndrome was first reported in the 1950s [5], and, as mentioned, typical features include facial asymmetry. However, ear–eye abnormalities are also observed. Moreover, vertebral anomalies and other congenital problems are likewise present. Further complexity of this syndrome is reflected by the frequent presence of concomitant issues, including severe OSA (obstructive sleep apnea) and sialorrhea [1,4]. Although several repetitive manifestations of the syndrome (e.g., hemifacial hypotrophy) exist, the condition may range from slight asymmetry to severe deformity. This helps explain why no rigid inclusion criteria are defined; diagnosis is based on clinical criteria. Diagnostic tools such as tomography, Magnetic Resonance Imaging (MRI), electroencephalography (EEG), ultrasound, and echocardiography aid in assessing symptom severity [2,5,6,7]. In addition, the syndrome is predominantly found in men [5]. Because the presentation of Goldenhar syndrome is highly variable, treatment procedures also differ. In many cases, surgical treatment is mandatory. This would include correction of eyelid colobomas and orthognathic surgery [4,7]. Auricular anomalies (observed in 80% of cases) may range from underdevelopment of the external ear to total hearing loss and may also require an interdisciplinary approach [7]. Besides esthetic concerns, individuals suffer from severe functional complications stemming from the existence of vertebral anomalies. The aforementioned account for head and neck movement limitations, which elucidate the reason for difficulties in laryngoscopy and intubation in these patients [8].

Sleep disorders are pervasive in today’s modern world. The relationship between insomnia, headaches (with specific reference to migraine and a tension-type headache called TTH), temporomandibular disorders, malocclusion, and obstructive sleep apnoea shows how advanced medicine has become, and the multifactorial background of OSA is increasingly being better understood [9,10,11]. Recently, the term COMISA (comorbid insomnia and sleep apnoea) has emerged to denote a syndrome characterized by the simultaneous presence of both conditions [9]. The definition of OSA is a recurrent upper airway obstruction during sleep, which is a life-threatening condition that results in intermittent hypoxia, sleep fragmentation, and sympathetic surges [9,10,12]. Findings by Emodi-Perlman et al. [13] revealed that OSA is also more frequently reported by men as compared to women. Upon awakening, patients with this condition may experience mild-to-moderate headaches. They are mostly bilateral and of the dull/pressure type [12]. In some cases, the headaches associated with OSA may mimic migraine or cluster headaches [14,15]. The aforementioned symptoms were originally thought to be caused by hypoxia-induced cerebral vasodilation; however, nowadays, the etiology remains unclear [16]. It is crucial to take into account that observations of poorer cognitive development and general growth in children suffering from breathing disorders have been noted. The consequence of a lack of treatment of these issues might result in cardiopulmonary complications, as well as behavioral disorders, such as hyperactivity, somatic complaints, and reduced attention when concentrating [17].

Multiple implications, coupled with the unclear origin of OSA, explain why modern medicine has taken a great interest in deeper investigation and research.

The PubMed, Web of Science, Scopus and Cochrane Library databases were searched by words and phrases of “OSA” (or “Obstructive sleep apnea”) and “Goldenhar syndrome”. The abstracts of the articles were screened for this topic. Due to the multitude of articles present (and their lack of uniformity), a narrative review was selected to best describe different patient cases. If the scientific background of the papers was not clear, they were rejected. The final search was performed in February 2026. More detailed data was described in the Section 2.

## 2. Materials and Methods

Studies were included if they were published in English:

- Concerned patients with Goldenhar syndrome (also known as oculo–auriculo–vertebral spectrum/ocular–auricular–vertebral syndrome);
- Discussed congenital, chromosomal, respiratory, airway, or breathing-related abnormalities connected to obstructive sleep apnea;
- Original articles, reviews, case reports, or case series were taken into account;
- The papers were published within the last 10 years.

The search was performed by two independent researchers and reviewed by a dental professional, a dental professor specializing in rare diseases.

## 3. Features of Goldenhar Syndrome

Because oculo–auriculo–vertebral sSpectrum (OAV) is one of the most common congenital defects involving the 1st and 2nd brachial arches, most of its features relate to the face [1,2,4]. The first four brachial arches begin to fuse in the 4th week of gestation [5]. Diagnosis is made at birth by identifying characteristic facial features. Among them, the most frequent features include facial asymmetry (hemifacial microsomia or maxillary and mandibular hypoplasia), which tends to enlarge over time, as well as ear and eye abnormalities [1,4]. The presence of orofacial clefts, most commonly cleft lip and/or palate, is also more prevalent than in healthy individuals [5]. The diversity of the clinical symptoms is rather extensive and begins with a barely noticeable facial asymmetry. On the other hand, severe craniofacial deformities requiring complicated surgical procedures for their resolution are also observed [2,6]. Facial asymmetry (caused by hemifacial hypotrophy) of varying intensity is observed in all cases. Auricular anomalies are also common, but they are found in ca. 80% of individuals only [18]. The presence of odontomas is also reported with greater frequency [5]. Absent or partially formed ears, often observed with middle or inner ear defects, may result in hearing impairment or loss [5]. Among ophthalmic symptoms, the diversity is much greater and affects ca. 90% of individuals. The most common appears to be upper eyelid coloboma, observed in more than 75% of individuals [4,6]. On the other hand, it has been reported that epibulbar choristoma is observed in ca. 90% of patients [18]. These are benign congenital tumors characterized by excessive overgrowth of epidermal appendages, sweat glands, and lacrimal glands. Furthermore, other tissues, including connective tissues, fat, skin, and neural tissues, can be affected [19]. Other less common ophthalmic involvements described in the literature include lipodermoids and limbal dermoids [6]. This, consequently, might lead to visual deficits [5]. In addition to those three, other congenital malformations, such as vertebral anomalies (present in 50% of cases, including congenital scoliosis) and sialorrhea, have also been described [1,4,5]. As is typical of syndromes with mandibular underdevelopment, Goldenhar syndrome characteristically presents with breathing problems, especially during sleep, with the most common being obstructive sleep apnea [4,6,20]. This could be due to an intensification of vertebral defects, including scoliosis. Irregularities in other visceral organs may be linked to dysfunction of the cardiac, renal, or nervous systems. These abnormalities can cause seizures, situs inversus, tetralogy of Fallot, and renal impairment (hypoplasia or agenesis). The presence of seizures, as well as hearing impairment, may lead to delayed speech development [5]. The most common features of the syndrome were collected in Table 1.

## 4. Molecular Basis of Goldenhar Syndrome

The etiology of the syndrome is more complex—beyond the genetic background, environmental factors also play a role [1,2,3]. The most likely gene responsible for the syndrome was found on chromosome 22q, but recent findings confirm that multiple genes are involved [3,4]. The cause is rather a developmental field defect resulting from a breakdown in molecular signaling, specifically the retinoic acid and notch pathways, which direct neural crest cells to form the face [21]. It has been proven that the risk of developing Goldenhar syndrome is elevated among mothers who use teratogenic substances during pregnancy. The most common examples of these teratogenic drugs include retinoic acid, cocaine, and tamoxifen, as well as alcohol use and prenatal infections (e.g., rubella or influenza) [5]. To fully confirm all such findings, more advanced clinical genetic testing protocols (e.g., chromosomal microarray or whole exome sequencing—WES) would be crucial.

The oldest theory on the molecular basis of Goldenhar syndrome is based on comparisons with genetic anomalies, especially involving the 22q and 14q chromosomes. Although 22q11.2 deletion is most often associated with DiGeorge syndrome, some patients may exhibit a Goldenhar-like phenotype [22]. Chromosome 14 has also been proposed as a candidate in Goldenhar syndrome, with particular attention to the 14q32 and 14q21 regions. These loci are thought to play key roles in craniofacial symmetry, and mutations affecting them may cause asymmetry characteristic of the condition [23]. Another gene mutation is described in individuals with Goldenhar-like features: a heterozygous variant of the ZYG11B gene [24]. The involvement of this gene in cell cycle regulation during early craniofacial development is evident. Knockdown of the zebrafish homolog affects craniofacial cartilage architecture and regulates the expression of other factors, such as the cartilage master regulator SOX6. Expression of the zebrafish homolog is also regulated by retinoic acid (a toxic molecule associated with clinical features of OAVS) [24,25]. Another genetic anomaly observed in OAVS is a mutation on chromosome 3q29, involving a microduplication or microdeletion of 9 genes in this region. These genes encode components of the Notch signaling pathway, which governs cell differentiation and tissue boundaries [26].

The other dysregulation refers to retinoic acid (RA). Retinoic acid is a powerful morphogen that can be influenced by environmental factors, such as maternal exposure to toxins. Such a situation may cause the pathway disruption and influence RA homeostasis. On the other hand, RA regulates the expression of homeobox (HOX) genes, which are the leading genes that act as a developmental “blueprint” determining the identity of branchial arches and are responsible for their proper development and binding. When this signaling is skewed, the structural identity of the jaws and ears is compromised. Any disruption of the homeobox domain may impair facial development [21,24,25,26]. It has been shown that retinoic acid influences mRNA expression and is crucial for regulating critical cell processes, including cell proliferation, death, and development [27].

At the molecular level, Goldenhar syndrome is considered a neurocristopathy. Cranial neural crest cells give rise to the bone, cartilage, and connective tissue of the face. The syndrome results from disruption of the proliferation, migration, or survival of these cells [28]. Regulatory genes, in this case, govern endoderm formation and neural crest cell development, thereby preventing proper formation of the branchial arches. Jaw morphogenesis during embryogenesis depends heavily on the development and growth of Meckel’s cartilage. The signals that influence chondrocyte proliferation in this section are not well understood. Neural crest cells (NCCs) or chondrocytes promote blood vessel growth. Consequently, the blood secretes factors that instruct and guide chondrocyte proliferation [29]. This thesis is supported by CBCTs and X-ray scans of patients, as mandibular artery dysgenesis is often observed among individuals with hemifacial microsomia and is closely related to jaw hypoplasia. The most probable pattern of this phenomenon involves NCCs and their derivatives, which support blood vessel growth, thereby indirectly influencing the developing jaw. For that reason, facial asymmetry can be present [29]. One of the genes identified in a significant linkage region was FBLN2. This has been shown to be the key factor in craniofacial cartilage development, and FBLN2 knockout zebrafish exhibit craniofacial malformations due to abnormal chondrocyte morphogenesis (including abnormal differentiation, apoptosis, and proliferation of NCCs). Another consequence is that this issue downregulates the bone morphogenetic protein (BMP) signaling pathway in the zebrafish model [28]. These findings highlight the importance of FBLN2 as a candidate gene for Goldenhar syndrome. A similar phenomenon could be observed in SF3B2, which is also implicated in impaired cartilage development [30]. Vascular disorders and diseases causing tissue hypoxia, leading to underdevelopment, may complicate the distinction between genetic and non-genetic molecular triggers. Regardless of genetically driven developmental issues, a localized vascular accident (hematoma) and a cascade of cell death (apoptosis) during embryogenesis may trigger unilateral underdevelopment. For that reason, further research and genetic findings are of paramount importance and should be pursued to identify the specific molecular basis of Goldenhar syndrome.

## 5. OSA in Goldenhar Syndrome

Obstructive sleep apnea (OSA) is a common sleep disorder in which the upper airway repeatedly collapses during sleep, causing pauses in breathing (apnea) or shallow breaths (hypopnea). The primary issue in OSA is excessive relaxation of the throat muscles during sleep. Common symptoms include loud snoring, breathing pauses during sleep, and gasping or choking upon waking. Dry mouth upon waking may also occur due to mouth breathing at night. These symptoms and breathing problems result in fragmented, low-quality sleep, lower blood oxygen levels, and daytime fatigue. The exact origins are not known, as the condition is highly complex. It is often associated with obesity, enlarged tonsils, or structural neck issues [9,10,31]. Common symptoms include loud snoring, gasping, and morning headaches. The genetic basis of OSA should also be considered [11]. If untreated, OSA may lead to high blood pressure, hyperinsulinemia, heart problems, stroke, and daytime sleepiness. Diagnosis is most frequently based on polysomnography (PSG). The apnea–hypopnea index (AHI) is used to categorize OSA into 3 stages: mild (5–15 events/h), moderate (15–30), and severe (>30) [22,31].

OSA during sleep is characterized by recurrent upper-airway obstruction. This condition causes intermittent hypoxia, sleep fragmentation, and severe health issues. It has been linked to hypertension, sympathetic activation, endothelial dysfunction, oxidative stress, and inflammation. Experimental and clinical literature also support intermittent hypoxia as a mechanism contributing to blood pressure elevation, including evidence from sleep-disordered-breathing models and human exposure studies [10,11].

The etiology of OSA in Goldenhar syndrome is primarily anatomical and multifactorial. It is reported in 7 to 67% of cases. The main issue is severe skeletal hypoplasia (micrognathia). In most cases, Goldenhar syndrome results in unilateral growth impairment. The impact on the airway is caused by the rotational deformity of the mandible during growth. Both mandibular and maxillary deficiencies restrict the oropharyngeal and nasopharyngeal spaces. In addition to the bone defect that affects breathing, glossoptosis (a posteriorly positioned tongue) and lymphoid hyperplasia (tonsils and adenoids) further compromise the airway [32].

An interesting case was described by American researchers [33], where OSA in a 4-year-old boy with Goldenhar syndrome was intensified due to nasal obstruction with the presence of an inverted tooth in the individual.

## 6. Treatment Methods

The most common treatment options among patients with Goldenhar syndrome are based on surgical procedures. Facial asymmetry can be resolved with orthognathic surgery using fibula-free flaps to reconstruct the mandible [20,34]. The surgery in this case is indeed more complex, but, as in most such cases, improvements after treatment are indisputable [35]. In addition to the difficulty of the procedure itself, vertebral anomalies may impair head and neck movements, thereby complicating laryngoscopy and intubation required for any surgical procedure [8]. The procedures are not based solely on facial asymmetry management but also focus on repairing other malformations, including auricular and ophthalmologic ones. One of the performed procedures is eyelid coloboma repair [7]. The management of bone malformations may result in improvement of breathing at night, however. The American Academy of Sleep Science [36] recommends that surgical interventions be considered a last-resort treatment option. This, however, could not apply to the specific cases of congenital diseases, especially with visceral cranium involvement. Examples of this phenomenon include Pierre Robin Sequence, Treacher Collins Syndrome, and Goldenhar syndrome [20]. For this reason, the treatment of such syndromes should combine non-surgical and surgical approaches, as a single treatment may be insufficient.

### 6.1. Continuous Positive Airway Pressure

Continuous positive airway pressure (CPAP) is the method of choice in non-surgical management of OSA in the general population. It is based on positive airway pressure ventilation, in which air is continuously delivered to the upper respiratory tract. The upper airway is prevented from collapsing by applying a pressure greater than atmospheric pressure. This method is generally used as first-line management in mild-to-moderate cases [20,29,37,38]. Further, it is generally effective in managing OSA and could be considered for use in individuals with Goldenhar syndrome as well. Possible challenges with treatment may be due to improper mask fit or the patient’s young age, which may also explain reduced or absent patient compliance [33,38]. Although the method yields good results in breathing management, 8% of individuals without congenital diseases stop treatment after the first night, and 50% quit within the first year of therapy. This might be because CPAP does not cure the origin of the problem, but rather helps with management [38]. The percentage of full recovery after the procedure is rather low in general, with a high tendency for this problem to occur in patients with congenital diseases such as Goldenhar or Treacher Collins syndromes [4,20,33]. Another alternative to surgical treatment for widening the upper airways is the use of nasal airway stents, introduced in the 1970s. The main limitation here is that, although successful, it can only be used temporarily. Moreover, it is merely a preliminary treatment performed prior to surgical correction. The stents could be used in small children, including newborns and neonates, with another indication for treatment of glossoptosis [20].

### 6.2. Mandibular Advancement

Mandibular advancement is a standard procedure for relieving the symptoms of OSA. For this purpose, specially constructed devices or orthodontic appliances, such as functional appliances, could be used. In general, mandibular advancement would reduce apnea and hypopnea. Diagnostic tools such as cephalograms and MRI show airway widening resulting from the correction of malocclusion. The widening of the airways in this case is closely connected to reducing the symptoms of night apnea, which makes this method sufficient to manage less severe cases, where the connection between malocclusion and the presence of breathing disorders is clear [10,39,40]. With mandibular advancement, the need for surgical intervention and mandibular elongation via bilateral sagittal split osteotomy (BSSO) might be avoided.

### 6.3. Surgery

Due to the asymmetry of the facial skeleton and soft tissues, a surgical procedure is often required to achieve an esthetic outcome. This involves the management of mandibular hypoplasia, as well as the midface asymmetry that had developed. The procedures are mainly performed after growth completion, but there may be exceptions, and surgeries can be performed earlier. Today, advancements in technology allow procedures to include virtual surgical planning to improve facial features, which can be accompanied by distraction osteogenesis or bone grafting, as well as temporomandibular joint (TMJ) reconstruction. In most cases, a bi-max procedure that includes LeFort I osteotomy and BSSO is performed. Bone grafting is used to correct deficiencies in the mandibular, zygomatic, or orbital regions [41,42]. A multidisciplinary approach is needed in these cases, and an experienced team is required. The craniofacial surgeon must plan the procedures with exceptional accuracy, taking into account abnormalities of the facial skeleton (e.g., absence of the mental foramina). A specialized surgeon, orthodontist, and anesthesiologist are essential to the success of these more complex treatment plans, underscoring the importance of establishing a multidisciplinary team of highly specialized experts [42,43].

According to the American Academy of Sleep Science [44], surgical management of OSA should be avoided if possible. Based on these recommendations, a non-surgical approach to managing this problem is strongly encouraged. However, for severe facial skeletal deformities, this strategy is not always feasible. As mentioned, surgical procedures are usually the key to adequate management of Goldenhar syndrome. Reconstruction of the facial skeleton with BSSO achieves mandibular lengthening. BSSO is typically performed in adult patients, whereas mandibular distraction osteogenesis (MDO) is more commonly used in growing individuals. Mandibular lengthening advances the base of the tongue and, consequently, opens the airways [41,42,45]. In addition to improving facial features and occlusion, both BSSO and MDO enhance airway width and reduce the AHI (apnea–hypopnea index). The post-treatment reduction in this index provides compelling evidence that the procedure helps alleviate sleep-disordered breathing, despite the persistence of mild OSA symptoms [46,47]. In life-threatening cases of OSA in patients with Goldenhar syndrome, the surgeon might decide to perform a tracheostomy to achieve immediate improvement in airway patency [48]. It is estimated that up to one quarter of individuals might need this procedure due to the syndrome’s high morbidity and negative impact on speech development. This is applied only to individuals when other methods cannot be used [49].

Another method of managing OSA is hypoglossal nerve stimulation (HNS or HGNS). This procedure is based on surgical access to the nerves in order to stimulate them. Although it is becoming more popular among healthy individuals, there has previously been a lack of data on its use in patients with this congenital defect [50,51]. The method was first applied to stimulate one side of the tongue and did not interfere with the soft palate and throat. Hypoglossal nerve stimulation reduces the risk of complete concentric collapse (CCC) of the velopharyngeal airway [52,53]. The results are promising, with an estimated success rate of 80% [10], but further studies are required. The procedure seems to be successful and, hopefully, will be incorporated in the near future into the treatment of patients with congenital defects.

There are multiple ways of managing OSA in patients with Goldenhar syndrome. Compared with healthy individuals, non-surgical management (e.g., continuous positive airway pressure) also reduces the apnea–hypopnea index (AHI) and improves oxygen saturation. In contrast to healthy individuals, though, OAVS patients require surgical mandibular distraction osteogenesis, which makes the management of OSA a single procedure for these patients. After that, polysomnography reveals improved SpO2 parameters, with SpO2 increasing from below 76% to above 95%, while the AHI decreases to less than 2 episodes per hour [54]. Additionally, the decrease in midfacial height resulting from facial underdevelopment is strongly correlated with an increased AHI in syndromic patients. This phenomenon also refers to other genetic syndromes, such as Down Syndrome, Crouzon Syndrome, and Pierre Robin Sequence [55].

The multitude of methods of managing OSA in patients with Goldenhar is extensive. To summarize, the most common procedures are provided in Table 2.

### 6.4. Perspectives

In contrast to the American Academy of Sleep Science recommendations [44] for managing OSA, surgical intervention appears to be the most optimal treatment for patients with congenital symptoms. In addition to improved facial features and mastication, an immediate improvement in daily activities and quality of life can be observed. Although surgical treatment and mandibular advancement might increase airway volume and therefore help manage OSA, this has not been demonstrated in long-term outcomes [10,56]. In most cases, patients require long-term orthodontic treatment with support from a multidisciplinary team. Managing OSA via mandibular advancement in such cases should be viewed as a supplementary treatment, secondary to the primary one, which is orthognathic surgery.

## 7. Discussion and Conclusions

Facial development begins in the fourth week of development with the branchial arches, which give rise to most craniofacial structures. The first arch forms the mandible, the muscles of mastication, the incus and malleus, and the maxillary and mandibular branches of the trigeminal nerve. The second branchial arch forms the stapes in the ear, the body and lesser horn of the hyoid, the facial nerve, and the muscles of facial expression [5]. Abnormal development of the 1st and 2nd branchial arches may result in Goldenhar syndrome, caused by disruption of mesodermal and neural crest cell migration or by abnormal embryonic vascular supply to the branchial arches during fetogenesis [5]. This anomaly was first described in 1952 in Switzerland [44]. The embryological background of the syndrome explains the multisymptomatic presentation. For this reason, management requires a multidisciplinary approach, ranging from CPAP to mandibular distraction, and should be tailored to the patient’s specific anatomy. Embriologically, development of the first and second branchial arches leads to impaired mandibular development and other anatomical malformations, such as maxillary hypoplasia and high-arched or clefted palates. Retrognathia displaces the tongue posteriorly, a condition called glossoptosis. This directly impairs the retroglossal airway. Additionally, while the maxillary and mandibular bones are hypoplastic, soft tissues (tongue and tonsils) maintain normal proportions, physically overwhelming the anatomically narrowed upper airway [57,58,59,60]. These defects reduce the cross-sectional area of the pharyngeal space, predisposing the patient to severe upper airway collapse, especially during sleep. This is the main issue with OSA. Additionally, disruptions in the embryonic vascular supply and neural crest cell migration might lead to neuromuscular weakness, which could also deepen upper airway collapse.

Treatment of Goldenhar syndrome requires multidisciplinary care and includes a combination of medical, supportive, and surgical therapies. From birth, the patient may need feeding assistance. Later, treatment usually includes hearing aids and glasses. To reduce the risk of developmental delays, physical and speech therapies are provided, and they show promising improvement in the individual’s development. Also, some advanced reconstructive and cosmetic surgeries should be performed, including cleft lip and palate repair, later dermoid excisions, and mandibular reconstruction [5]. Understanding the origins of OSA in patients with Goldenhar syndrome also helps manage the problem.

Although the relationship between OSA and malocclusion is debatable [9], the need for orthodontic treatment in society is, in most cases, driven by increased esthetic expectations regarding the dentition [61]. Among patients with congenital defects, the need for orthodontics usually precedes preparation for orthognathic surgery procedures. Generally, improvements in overall health and quality of life have been reported after orthognathic surgery [62]. The specificity in Goldenhar syndrome is that unilateral underdevelopment of the mandible can lead to unilateral airway constriction. This is due to uneven muscular development and the resulting lack of support for the anatomical structures of the airways [63]. Improvement in the reduction of OSA syndromes was also observed among patients with Goldenhar syndrome after orthognathic procedures. In most cases, bimaxillary orthognathic surgery was found to be insufficient; therefore, more advanced procedures with bone grafting had to be performed. The use of a fibula free flap for mandibular reconstruction should be considered a viable treatment option for the management of OSA in individuals with Goldenhar syndrome [32]. A facial artery musculo-mucosal (FAMM) flap could also be considered to shorten the surgical procedure [34]. The specificity of treatment in this case is that the underdevelopment is structural (e.g., underdeveloped jaws) and not limited to the soft tissues. Management requires personalized procedures, most frequently based on craniofacial surgery. This management requires individual treatment planning in the first place. Also, the procedures are more complicated, so an experienced treatment team would be beneficial. 

This paper was constructed in order to highlight the most common procedures used to manage OSA in patients with Goldenhar syndrome, but its greatest limitation is that there is no standardized method of managing the problem in patients with congenital diseases. This underscores the paramount importance of developing and advancing this type of procedure in the future.

Management of OSA in Goldenhar syndrome requires early screening of the patients. It is recommended to perform polysomnography in all such patients and, if possible, repeat it each year. Treatment should be coordinated by the craniofacial team (with the maxillofacial surgeon as the team leader, accompanied by an otorhinolaringolodist, a pulmonologist, and an orthodontist). Long-term monitoring requires post-treatment PSG, which must be repeated annually to confirm resolution of OSA, as residual apnea is common.

## Figures and Tables

**Table 1:** Most common manifestations of Goldenhar syndrome [1,2,4,5,6,18,19,20].

Sitio	Syndrome
Face	Asymmetry of different intensity, hemifacial hypotrophy, microsomia, maxillary and mandibular hypoplasia, odontomas, cleft lip and/or palate
Ear	Auricular underdevelopment, middle or inner ear defects, hearing impairment or loss
Eye	Epibulbar choristoma, upper eyelid coloboma, lipodermoid, limbal dermoid, visual deficits
General	Congenital scoliosis, breathing impairment, OSA; affects cardiac (tetralogy of Fallot, situs inversus), renal (hypoplasia or agenesis) and nervous system; impeded speech development

**Table 2:** Management of OSA in Goldenhar patients.

Intervention	Effectiveness and Clinical Outcomes
**Adenotonsillectomy**	Often the first-line surgical treatment. While it improves AHI, it is rarely curative in Goldenhar patients due to underlying skeletal issues.
**Mandibular Distraction (MDO)**	A highly effective surgical procedure that gradually lengthens the jaw. Evidence shows significant increases in airway volume and high success rates in avoiding or removing tracheotomies.
**Positive Airway Pressure (CPAP)**	Effective for stabilizing the airway non-invasively, though long-term compliance is often hampered by craniofacial asymmetry and mask-fit issues.
**Orthodontic Intervention**	Maxillary expansion and functional appliances can serve as adjuncts in milder cases or as part of long-term surgical planning.

## Data Availability

No new data were created or analyzed in this study. Data sharing is not applicable to this article.

## Abbreviations

OAVS	ocular–auricular–vertebral syndrome
OSA	obstructive sleep apnea
MRI	magnetic resonance imaging
EEG	electroencephalography
TTH	tension-type headache
COMISA	comorbid insomnia and sleep apnoea
RA	retinoic acid
NCC	neural crest cell
HOX	homeobox (genes)
BMP	the bone morphogenetic protein
AHI	apnea–hypopnea index
PSG	polysomnography
CPAP	continuous positive airway pressure
BSSO	bilateral sagittal split osteotomy
MDO	mandibular distraction osteogenesis
HNS or HGNS	hypoglossal nerve stimulation
CCC	complete concentric collapse
FAMM	facial artery musculo-mucosal
